# Assessing Serious Spinal Pathology Using Bayesian Network Decision Support: Development and Validation Study

**DOI:** 10.2196/44187

**Published:** 2023-10-03

**Authors:** Adele Hill, Christopher H Joyner, Chloe Keith-Jopp, Barbaros Yet, Ceren Tuncer Sakar, William Marsh, Dylan Morrissey

**Affiliations:** 1 Electronic Engineering and Computer Science Queen Mary University of London London United Kingdom; 2 Bart's Health National Health Service Trust London United Kingdom; 3 Department of Cognitive Science Graduate School of Informatics Middle East Technical University Ankara Turkey; 4 Department of Industrial Engineering Hacettepe University Ankara Turkey; 5 Sport and Exercise Medicine Queen Mary University of London London United Kingdom

**Keywords:** artificial intelligence, back pain, Bayesian network, expert consensus

## Abstract

**Background:**

Identifying and managing serious spinal pathology (SSP) such as cauda equina syndrome or spinal infection in patients presenting with low back pain is challenging. Traditional *red flag* questioning is increasingly criticized, and previous studies show that many clinicians lack confidence in managing patients presenting with red flags. Improving decision-making and reducing the variability of care for these patients is a key priority for clinicians and researchers.

**Objective:**

We aimed to improve SSP identification by constructing and validating a decision support tool using a Bayesian network (BN), which is an artificial intelligence technique that combines current evidence and expert knowledge.

**Methods:**

A modified RAND appropriateness procedure was undertaken with 16 experts over 3 rounds, designed to elicit the variables, structure, and conditional probabilities necessary to build a causal BN. The BN predicts the likelihood of a patient with a particular presentation having an SSP. The second part of this study used an established framework to direct a 4-part validation that included comparison of the BN with consensus statements, practice guidelines, and recent research. Clinical cases were entered into the model and the results were compared with clinical judgment from spinal experts who were not involved in the elicitation. Receiver operating characteristic curves were plotted and area under the curve were calculated for accuracy statistics.

**Results:**

The RAND appropriateness procedure elicited a model including 38 variables in 3 domains: risk factors (10 variables), signs and symptoms (17 variables), and judgment factors (11 variables). Clear consensus was found in the risk factors and signs and symptoms for SSP conditions. The 4-part BN validation demonstrated good performance overall and identified areas for further development. Comparison with available clinical literature showed good overall agreement but suggested certain improvements required to, for example, 2 of the 11 judgment factors. Case analysis showed that cauda equina syndrome, space-occupying lesion/cancer, and inflammatory condition identification performed well across the validation domains. Fracture identification performed less well, but the reasons for the erroneous results are well understood. A review of the content by independent spinal experts backed up the issues with the fracture node, but the BN was otherwise deemed acceptable.

**Conclusions:**

The RAND appropriateness procedure and validation framework were successfully implemented to develop the BN for SSP. In comparison with other expert-elicited BN studies, this work goes a step further in validating the output before attempting implementation. Using a framework for model validation, the BN showed encouraging validity and has provided avenues for further developing the outputs that demonstrated poor accuracy. This study provides the vital first step of improving our ability to predict outcomes in low back pain by first considering the problem of SSP.

**International Registered Report Identifier (IRRID):**

RR2-10.2196/21804

## Introduction

### Background

Serious spinal pathologies (SSPs) are rare [1], difficult to identify [2], and have potentially life-changing implications for patients with low back pain (LBP). Examples include, but are not limited to, cauda equina syndrome (CES), spinal malignancies, spinal infections, and vertebral fractures. In clinical practice, *red flag* questions [3] are routinely used by health care professionals during their assessment to attempt to assess whether a suspicion of an SSP might be raised; however, many patients will report ≥1 of these symptoms without having a serious underlying pathology [4]. A number of studies have questioned the validity of red flags as indicators [2,4]. The red flag questions represent a complex reasoning algorithm that takes place in the decision-making process on the diagnosis or treatment pathway. A recent study exploring the use of clinical decision support to manage LBP in community pharmacies revealed clinicians’ uncertainty when screening for SSP [5]. Therefore, developing effective decision support to aid clinicians in the reasoning process is desirable; for example, a clinical decision support system (CDSS) for predicting the need for surgical intervention in patients with LBP has been implemented with some success in the Netherlands [6], but the authors of the study [6] have identified that the results could be improved with a Bayesian network (BN) technique [7]. BNs are capable of using expert opinion combined with evidence where it is available to give probabilistic outcomes for a given scenario [8]. BNs are graphical models that represent the probability distribution and conditional independencies among variables by a directed acyclic graph. BNs are ideally suited to reasoning under uncertainty because the conditional independencies encoded in their structure are used to compute complex probabilistic queries in an efficient way. Their graphical structure offers a suitable medium to encode domain knowledge. They can be combined with machine learning algorithms to learn from data.

A BN has been used to predict mental health conditions; however, this study relied upon a large data set (>17,000 data points) over multiple time points [9]. Indeed, the availability of large data sets containing outcome measures and a relatively low rate of errors is usually needed in the majority of development approaches used. Musculoskeletal medicine does not typically have the luxury of such data. As such, evidence, patient data, and expert opinion will need to be combined to replicate the reasoning process in musculoskeletal and spinal conditions, as demonstrated by Sheng et al [10].

### Objectives

**Figure 1:**
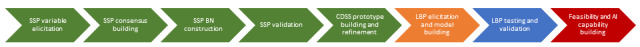
A design pipeline representing work completed as part of this study and future work toward assessing the feasibility of using a clinical decision support system (CDSS) in the assessment of serious spinal pathology (SSP) and developing its artificial intelligence (AI) capability. LBP: low back pain; SSP BN: serious spinal pathology Bayesian network.

The aim of our study was 2-fold: first, to develop and build a BN using a 3-stage elicitation, and second, to establish the accuracy of the BN following a robust validation framework. The remainder of this paper will discuss the development of these 2 aims, referring to the “BN.” We use this term intentionally to focus attention on the knowledge-based artificial intelligence (AI) sitting behind a potential CDSS.We consider that a CDSS comprises: a user interface, a software package to integrate with an electronic health record and an underlying knowledge-based AI model (the BN). As such the elements of the CDSS require separate development studies (Figure 1). The design and usability of a CDSS are beyond the scope of this study.

## Methods

### Building the BN

To be able to build the BN and represent relationships between the reported symptoms, risk factors, and potential outcomes, a formal elicitation process was needed to determine the variables to be included within the model. The considered approaches for the elicitation included a Delphi study [11], a RAND appropriateness method [12], and a focus group method [13]. After running an internal pilot with clinical colleagues [14], we deemed the RAND method to be the most appropriate. An outline of the elicitation procedure is provided in the *Elicitation Process* subsection. This paper focuses on the health care application of this method to SSP in LBP. For a full report of the mathematics behind the elicitation procedure and construction of the BN, the interested reader is directed to our protocol [14] and Multimedia Appendix 1 [8,12,14-20].

### Ethical Considerations

Ethics approval was granted by the Queen Mary University of London Ethics of Research Committee (QMREC2018/48 027). Informed consent was obtained from all participants in the study. All participant contributions were anonymized in the data collection. Participants were offered reimbursement of expenses and a shopping voucher in exchange for participation.

### Elicitation Process

The elicitation consisted of 3 distinct stages. In the first stage (variable elicitation), the 16 clinical experts considered which variables should be included in the model. In the second stage (structure elicitation), we asked the experts to link the variables together via the relationships between the variables to form the structure of the model. The third stage involved a probability elicitation to populate the model with appropriate values.

**Figure 2:**
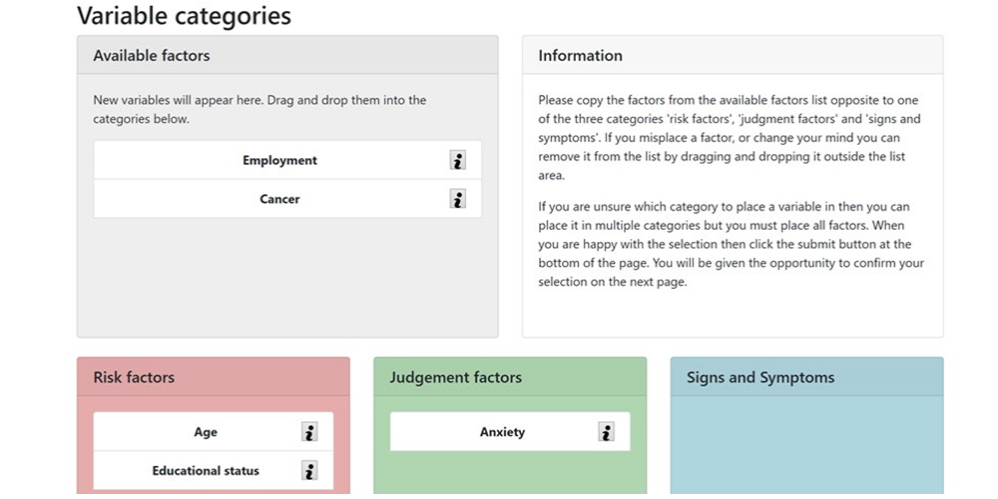
Screenshot of the web-based elicitation tool. The available variables are given in the gray box. Participants had the ability to drag the boxes to each category and add new variables. Participants were asked to rank the variables in each category in order of importance to the Bayesian model or clinical reasoning process.

**Figure 3:**
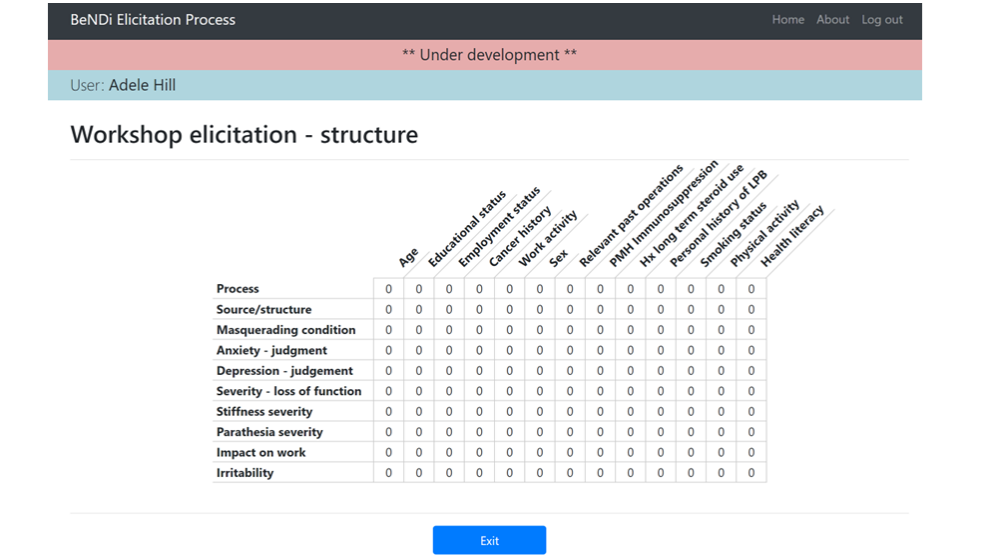
Screenshot of the stage 2 elicitation interface. Grids were provided to participants to determine the relationships between the variables in each category. This grid elicits the relationship between risk factors (columns) and judgment factors (rows). Hx: history; LBP: low back pain; PMH: past medical history.

**Figure 4:**
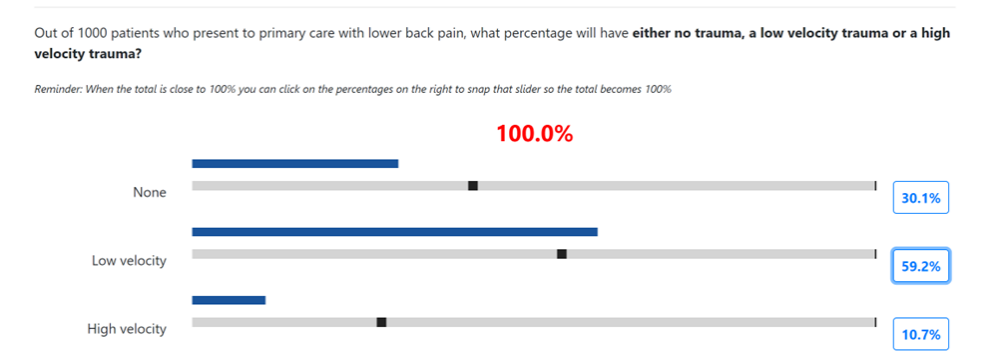
Screenshot of the stage 3 elicitation interface. The probability elicitation questions were constructed for the web-based interface. Here, the participant is required to provide a prior probability of a patient experiencing trauma by considering a large population of patients and providing an estimate based on their experience.

A web-based tool was developed to conduct the elicitation (Figures 2-4). The elicitation was based on an imposed structure of medical reasoning: risk factors, signs and symptoms, and judgment factors. The term *judgment factors* was used instead of *diagnosis* because it would be unrealistic to claim that this tool could reach a diagnosis. Rather, we aimed to produce a risk assessment or judgment as to what may be influencing the patient’s presentation.

Participants were asked to consider a patient scenario and decide what factors they would consider when assessing the patient and deciding on the next course of action. They were asked to consider *risk factors*, *signs and symptoms*, and *judgment factors* with respect to clinical reasoning, with explanations of each provided as part of the tool. A small number of factors were provided for them as an illustration (Figure 2).

Participants were able to use the tool (Figure 2) to drag and drop the provided variables into any category they deemed appropriate as well as add their own recommendations for variables. They could then rank the variables within each category to signify the importance of their inclusion in the resulting BN. During the web-based phase, this work was conducted individually. During the workshop, participants had the opportunity to discuss the variables in each category before revisiting the elicitation tool and completing the task again. In stage 2, participants used grids (Figure 3) to express how they believed the variables should be related to each other (ie, establishing the edges in the BN), which we used to form the structure of the BN. Participants entered a number ranging from 0 to 3 to signify the strength of the relationship, with 0 representing no relationship and 3 representing a strong relationship. Again, participants worked individually on the web, then came together in a workshop to form consensus. Consensus was calculated by taking the median scores and calculating the interpercentile range [12]. Variables with a high median score (ie, most of the participants thought that there was a strong relationship) and a low interpercentile range (ie, there was consensus) were included and formed an edge in the BN.

Stage 3 comprised a web-based elicitation only. The participants were guided through a series of questions to elicit the probabilities of events in the BN. An illustrative example can be found in Figure 4. Participants were given example scenarios with a clinical context and asked to estimate the probability of the scenarios. This facilitated the elicitation of partial node probability tables, with a plan to refine these with machine learning at a later stage.

### Model Validation

A basic validation of the resultant model was planned. Typically, in a machine learning algorithm, a training set and a test set of data would be used to validate the model. However, this is not possible owing to the constraints on the available data in musculoskeletal conditions. Therefore, to test the model, the framework for validating an expert-elicited BN proposed by Pitchforth and Mengersen [21] was used to conduct some preliminary model testing. The results of validating the model will help us plan for the next stages of work, that is, identifying what further development may be needed on the model.

The validation framework used is summarized in Table 1. This framework recommends 7 domains of validation. In this study, it was possible to use 4 (57%) of the 7 domains; the domains *concurrent*, *convergent*, and *discriminant* require a comparator model; however, no appropriate alternative SSP model could be found. Further work is therefore required to identify a suitable comparator model in an alternative field of medical diagnosis. The methods used for the other 4 domains are detailed in Table 1.

**Table 1:** Validation framework.

Validation type	Brief summary	How it was done
Nomological	Does the model fit within an appropriate context in the literature?	Comparison with the literature
Face	Does the model structure look the same as experts of the literature predict?	Peer review with spinal experts
Content	Does the model structure contain all and only the factors and relationships relevant to the model output?	Peer review with spinal experts
Predictive	Is the model behavior predictive of the system being modeled?	Literature case studies and real-life cases
Concurrent	Does the model structure act identically to a network modeling a theoretically related construct?	For future validation
Convergent	How similar is the model structure to other models that are nomologically proximal?	For future validation
Discriminant	How different is the model structure from other models that are nomologically distant?	For future validation

### Nomological Validation

The judgment factors that had been clearly defined by the participants were CES, space-occupying lesion, fracture, infective condition, inflammatory condition, and nerve root condition (encompassing radiculopathy and radicular pain from L1 to S1). As such, it was possible to find published guidance on the signs and symptoms as well as risk factors for nomological validation. Cord compression was not defined well enough and could have been construed as a sequela of the other pathologies listed. Irritability of pain, depression, stress, and anxiety were only examined in relation to a patient with LBP, rather than in terms of the medical diagnosis of these conditions. Therefore, it was not appropriate to compare these disorders with published diagnostic criteria because the participants did not intend to diagnose them; they only wanted to identify the disorders’ impact on LBP or symptoms.

The research literature was explored for guidelines and consensus statements on cauda equina, space-occupying lesion, fracture, infective condition, inflammatory condition, and nerve root condition. Comparison was made with the recent consensus study by Finucane et al [22] on the variables *cauda equina*, *space-occupying lesion/cancer (malignancy)*, *fracture*, and *infective condition (spinal infection)*. For *inflammatory condition*, comparison was made with the study by McCrum [23]. For *nerve root condition*, comparison was made with the National Institute for Health and Care Excellence (NICE) guidelines for lumbar radiculopathy [24]. Each paper identified a series of clinical signs and symptoms as well as risk factors for the conditions.

### Face Validation

Five spinal experts were recruited via convenience sampling. The experts did not participate in the initial elicitation or in any other aspect of the study. The model structure was presented to the experts using a web-based tool [15] and an explanation of what a BN is. The experts were given the opportunity to explore the structure of the BN independently and comment as they felt necessary.

### Content Validation

The spinal experts were asked to review the model and comment on the appropriateness of individual variables and the appropriateness of connections among the variables as well as suggest any new variables or relationships that they thought were missing from the model.

The Gwet agreement coefficient 2 test [25] for agreement was used to assess the responses of the spinal experts compared with those of the original study participants. The results were interpreted using the Landis and Koch criteria [26].

### Predictive Validation

A series of patient case studies were taken from the literature [22,27] and clinical practice, with known outcomes. The cases, which are summarized in Table 2, were used to make predictions using the resulting model and compared with the actual outcomes.

**Table 2:** Cases taken from the literature and clinician experience^a^.

Baseline case	Study, year	Variations to baseline case for sensitivity analysis
Low-risk CES^b^	Finucane et al [22], 2020	Higher risk (1 additional symptom) Additional symptoms of cancer Additional symptoms of inflammatory condition
Low- to midrisk CES	Finucane et al [22], 2020	Higher risk (1 additional symptom) Additional symptoms of cancer Additional symptoms of inflammatory condition
Midrisk CES	Finucane et al [22], 2020	Higher risk (1 additional symptom) Additional symptoms of cancer Additional symptoms of inflammatory condition
High-risk CES	Finucane et al [22], 2020	Higher risk (1 additional symptom) Additional symptoms of cancer Additional symptoms of inflammatory condition
Low-risk fracture	Finucane et al [22], 2020	Higher risk (1 additional symptom) Additional symptoms of cancer Additional symptoms of inflammatory condition
Low- to midrisk fracture	Finucane et al [22], 2020	Higher risk (1 additional symptom) Additional symptoms of cancer Additional symptoms of inflammatory condition
Midrisk fracture	Finucane et al [22], 2020	Higher risk (1 additional symptom) Additional symptoms of cancer Additional symptoms of inflammatory condition
High-risk fracture	Finucane et al [22], 2020	Higher risk (1 additional symptom) Additional symptoms of cancer Additional symptoms of inflammatory condition
Low-risk malignancy	Finucane et al [22], 2020	Higher risk (1 additional symptom) Additional symptoms of inflammatory condition
Low- to midrisk malignancy	Finucane et al [22], 2020	Higher risk (1 additional symptom) Additional symptoms of inflammatory condition
Midrisk malignancy	Finucane et al [22], 2020	Higher risk (1 additional symptom) Additional symptoms of inflammatory condition
High-risk malignancy	Finucane et al [22], 2020	Higher risk (1 additional symptom) Additional symptoms of inflammatory condition
Low-risk infection	Finucane et al [22], 2020	Higher risk (1 additional symptom) Additional symptoms of cancer Additional symptoms of inflammatory condition
Low- to midrisk infection	Finucane et al [22], 2020	Higher risk (1 additional symptom) Additional symptoms of cancer Additional symptoms of inflammatory condition
Midrisk infection	Finucane et al [22], 2020	Higher risk (1 additional symptom) Additional symptoms of cancer Additional symptoms of inflammatory condition
High-risk infection	Finucane et al [22], 2020	Higher risk (1 additional symptom) Additional symptoms of cancer Additional symptoms of inflammatory condition
Inflammatory condition	Real case	Higher risk (1 additional symptom) Additional symptoms of cancer
Inflammatory condition	Real case	Higher risk (1 additional symptom) Additional symptoms of cancer
Nerve root condition	Jones and Rivett [27], 2018	Higher risk (1 additional symptom) Additional symptoms of cancer Additional symptoms of inflammatory condition

^a^Cases were modified as described for sensitivity analysis and assessed by an independent third party.

^b^CES: cauda equina syndrome.

## Results

### Elicitation

**Figure 5:**
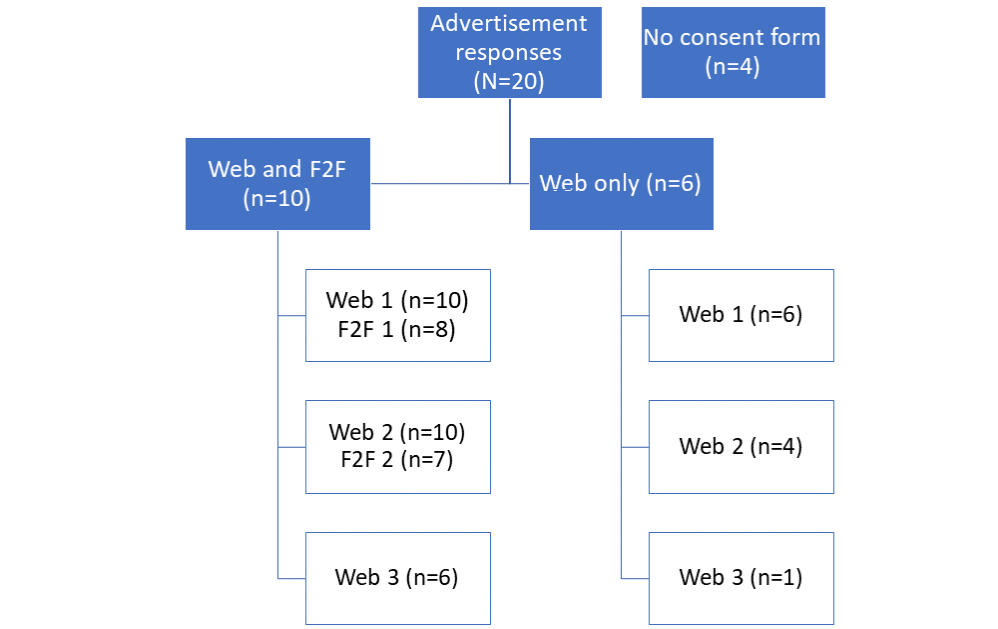
Study recruitment and participation. F2F: face-to-face workshop.

Sixteen participants were recruited to the study (Figure 5). In stage 2, those who failed to complete the web-based elicitation before the study deadline (8/16, 50%) were excluded from further participation. The remaining participants (8/16, 50%) attended the first face-to-face workshop. Of these 8 participants, 7 (88%) attended the second face-to-face workshop, whereas 1 (12%) dropped out. Participant numbers between 7 and 15 are recommended [12].

### Formulating a BN

**Figure 6:**
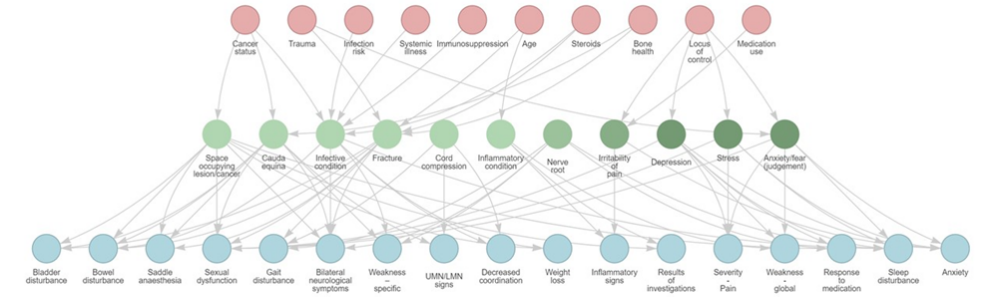
The Bayesian network that resulted from the 3 stages of the study. The pink row represents risk factors, the green row represents judgment factors, and the blue row represents signs and symptoms (see Figure 2). The arrows are formed from the grid relationships elicited in stage 2. The arrows represent causal relationships; for example, “Cancer status” increases the probability of being diagnosed with space-occupying lesion/cancer, and having cancer increases the probability of having the signs and symptoms associated with space-occupying lesion/cancer. LMN: lower motor neuron; UMN: upper motor neuron.

A BN was formulated from the results of the 3 stages of the study. The risk factor variables (pink) and signs and symptoms variables (blue) in Figure 6 represent the specific questions that require answers to produce a probability of a condition (green). For variables with no user input, the BN will accept *unknown* and use a prior baseline assumption.

Participants were asked to list the variables pertinent to patients presenting with LBP and rank them in order of importance. During the 2 face-to-face workshops, participants were clear on the need to explore the role of spinal red flags and exclude SSP before any other aspect of LBP. They produced a list of categories and expressed how they believed the elicited variables should sit within these categories. In the second workshop, these categories were used to produce 2 grids. In the grids, participants gave a value ranging from 0 to 3 to denote the strength of the relationship between the variables in the rows and the variables in the corresponding columns. This yielded a clear delineation in the results: SSPs were consistently scored very highly, with a low interpercentile range, and all other factors received a low score. As such, the resulting BN primarily contains variables and edges associated with SSP.

### Validation

#### Nomological Validation

Table 3 contains the symptoms and risk factors identified in the cited study for the respective condition.

It is worth noting that unilateral leg pain and paresthesia were factors that were initially included in stage 1 of the process; however, they were not ranked highly enough in the subsequent rounds to be included in the BN for CES.

Sleep disturbance is a factor contained in the BN but is not linked to the space-occupying lesion/cancer variable. Balance issues were not identified as a factor by participants.

Some of the factors identified for fracture are contained within the BN but not linked to fracture. Factors such as sex and smoking history were identified in our study but excluded by the ranking exercise. There were factors such as pain lying supine and early menopause or late menarche, which were never identified in our study.

Smoking and obesity were factors identified in stage 1 of this process for spinal infection risk; however, these factors did not score highly enough in the later stages to make it into the final BN.

For inflammatory condition, improvement with movement and family history are not contained in the BN and were not identified at any stage by our participants. Improvement with nonsteroidal anti-inflammatory drugs and waking at night (ie, sleep disturbance) are contained within the BN but did not score highly enough to be linked to inflammatory condition.

All factors listed were identified in our study in the early stages for nerve root condition. However, owing to the focus on SSP, the final scores attained did not allow for these factors to make it into the final BN.

**Table 3:** Factors associated with the spinal pathologies featured in the Bayesian network (BN) as taken from Finucane et al [22] (cauda equina syndrome, spinal infection, fracture, and malignancy), McCrum [23] (inflammatory condition), and National Institute for Health and Care Excellence (NICE) guideline 59 [24] (nerve root condition).

Factors associated with spinal pathology			Contained in BN
**Cauda equina syndrome [22]**			
	Unilateral radicular pain		
	Bilateral radicular pain	✓	
	Dermatomal reduced sensation		
	Myotomal weakness	✓	
	Changes to bladder function	✓	
	Changes to bowl function	✓	
	Saddle sensory disturbance	✓	
**Malignancy [22]**			
	History of cancer	✓	
	Night pain		
	Requiring strong pain killers	✓	
	Weight loss	✓	
	Balance issues		
	Odd sensations in legs	✓	
**Fracture [22]**			
	History of cancer		
	Multiple myeloma		
	Osteoporosis	✓	
	Severe pain or worsening pain		
	Age	✓	
	Sex		
	Smoker		
	Pain worse when lying supine		
	Early menopause or late menarche		
	History of fracture		
**Spinal infection [22]**			
	Immunosuppression	✓	
	Steroid use	✓	
	Smoking		
	Obesity		
	History of TB^a^	✓	
	Fever	✓	
	Neurological dysfunction	✓	
	User of intravenous drugs	✓	
	Night pain	✓	
**Inflammatory condition [23]**			
	Age	✓	
	Waking at night		
	Improvement with movement		
	Improvement with NSAIDs^b^		
	Past enthesitis, psoriasis, or arthritis	✓	
	Family history		
	Uveitis	✓	
	Inflammatory bowel disease	✓	
**Nerve root condition [24]**			
	Unilateral leg pain		
	Dermatomal changes		
	Myotomal weakness	✓	
	Straight leg raise test positive		
	Age		
	Smoking		
	Obesity		

^a^TB: tuberculosis.

^b^NSAID: nonsteroidal anti-inflammatory drug.

#### Face and Content Validation

The agreement statistics (Gwet agreement coefficient 2 test) are summarized in Table 4, and the Landis and Koch [26] interpretation of agreement statistics is presented in Table 5. There was *almost perfect* agreement between the original study participants and the independent spinal experts for 9 (82%) of the 11 judgment variables in the BN. Some confusion over the term *irritability of pain* led to the agreement being reduced to *substantial*. Issues identified with the connections to the fracture variable reduced the agreement to *fair*.

**Table 4:** Summary of agreement analysis between participants and spinal experts.

Preexisting variables	Gwet agreement coefficient 2	Agreement level
Space occupying lesion	1	Almost perfect
Cauda equina syndrome	0.85	Almost perfect
Infective condition	0.86	Almost perfect
Fracture	0.34	Fair
Cord compression	1	Almost perfect
Inflammatory condition	0.91	Almost perfect
Nerve Root	0.93	Almost perfect
Irritability of pain	0.68	Substantial
Depression	1	Almost perfect
Stress	1	Almost perfect
Anxiety	0.95	Almost perfect

**Table 5:** Landis and Koch [26] interpretation of agreement statistics.

Agreement statistics	Interpretation
<0	Poor
0-0.2	Slight
0.21-0.4	Fair
0.41-0.6	Moderate
0.61-0.8	Substantial
0.81-1	Almost perfect

#### Predictive Validation

**Figure 7:**
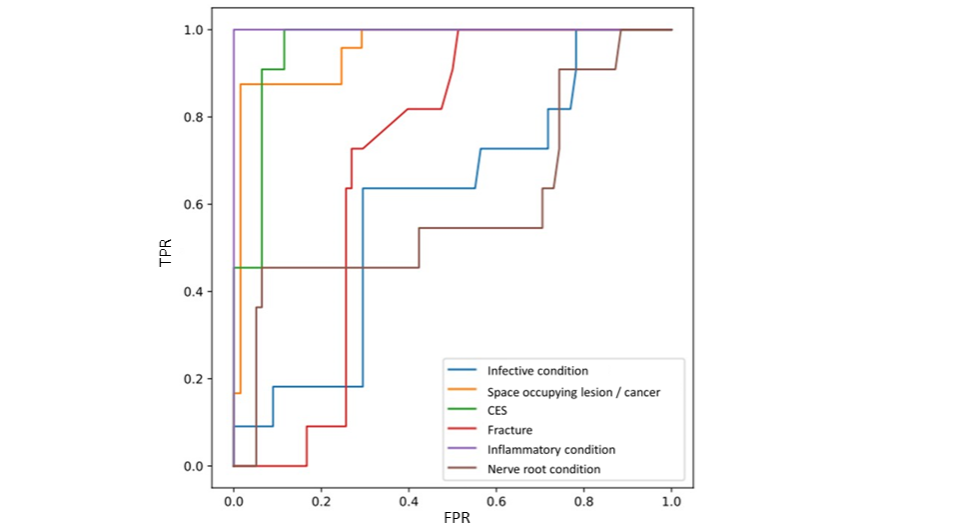
Receiver operating characteristic curves (area under the curve values) for patient scenarios. CES: cauda equina syndrome; FPR: false positive rate; TPR: true positive rate.

The cases described in Table 3 were codified to produce inputs for the BN, and a corresponding label of 0 (no) or 1 (yes) was attached for each of the 6 possible conditions. The labels were compared against the probabilistic outputs of the BN to produce receiver operating characteristic curves (Figure 7). The area under the curve (AUC) was calculated, with perfect predictions having an AUC value of 1 and poor predictions having an AUC value of near 0.5.

Good predictive capabilities were shown for CES (AUC=0.96) and space-occupying lesion/cancer (AUC=0.96), although the perfect results for inflammatory condition (AUC=1.0) should be taken with caution owing to very few instances within the case list. Fracture (AUC=0.7), infective condition (AUC=0.6), and nerve root condition (AUC=0.59) fared less well in their predictive values.

#### Masquerading Conditions: Clinical Implications

The BN demonstrated excellent validity for CES, inflammatory condition, and space-occupying lesion/cancer across 4 domains of validation.

The BN scored less well with suspected spinal fractures on nomological, face, and content validity; upon further inspection, the risk factor variables connected to fracture within the BN were *trauma*, *bone health*, *steroids*, and *age*. The signs and symptoms were related to *bladder and bowel disturbance*, *saddle anesthesia*, *sexual dysfunction*, and *bilateral neurological symptoms*. On reviewing the recorded discussions, we observed that there was a level of nuance around traumatic versus pathological fractures, which has not been captured in the final model structure. A link has not been made by the experts to the following key variables identified by Finucane et al [22]: pain levels, sleep disturbance, alcohol intake, and cancer history. Likewise, Finucane et al [22] did not identify bladder, bowel, saddle anesthesia, or gait disturbance as indicative of spinal fracture. This highlights a potential weakness in the BN structure elicitation. It is not possible for a BN model to contain all variables and all relationships because this would increase the size of the model, leading to increased prediction times, which would subsequently affect its deployability in clinical practice [28]. It also emphasizes the importance of not relying solely on one group of experts or one source for knowledge elicitation.

Similarly, for nerve root condition, only 1 (14%) of the 7 factors identified in the NICE guidelines [24] was chosen to represent this pathology in the model in its nomological validity. It scored reasonably well in terms of predictive validity and face validity; however, the lack of nomological validity may have contributed to its lower accuracy. This was due to the focus on SSP influencing the ranking scores in the later rounds.

Although infective condition performed well in nomological, face, and content validity, the predictive validity was not as accurate as other conditions. This perhaps reflects the difficulty in assessing for this condition because the signs and symptoms are very similar to those of other conditions, and the risk factors are rare.

It is unclear whether a separate variable for *cord compression* is required. There is a paucity of literature on cord compression that does not result from either CES, cancer or metastatic spinal cord compression, or a spinal fracture, which are all contained separately in the model. We were unable to complete the full complement of validation exercises for this condition for that reason.

Irritability of pain, stress, depression, and anxiety are not, strictly speaking, serious underlying pathologies, although they are very important considerations to make when assessing spinal pain. Because of the focus of the original participants on identifying SSPs, these variables are less well served by the risk factors and signs and symptoms that were able to be contained in the constrained BN. As such, it is difficult to justify their inclusion in this model. It is likely that further work on a complementary model would be required to do justice to the assessment of stress, anxiety, and depression and their role in LBP.

## Discussion

This process has allowed us to develop a BN using a 3-stage elicitation process with clinical experts. We have also established the areas of the resulting BN that have achieved good levels of accuracy and identified those that will require further work by using a robust validation framework.

### Building a BN Model

The 3-stage RAND process has allowed us to build a BN model that provides a prediction of SSP. The expertise of the participants in this study centered around spinal masquerading conditions, and the perception that these were the *not-to-be-missed* pathologies were judged to be key to the success of the model.

Our study has followed a well-trodden path of manually building a BN using expert-elicited data [28,29]. The rise in advanced automated machine learning techniques has led to a decline in the more time-consuming manual building approaches. However, this manual method is suited to the problem of SSP owing to the lack of structured data [8]. Moreover, the deep integration of experts within the construction process helps engender trust in the model, which is critical to its success [30]. We completed the elicitation procedure with relatively low time burden on the experts by using a bespoke web-based elicitation tool, thereby negating one of the main disadvantages of the manual method. This also had the advantage of eliciting a range of views and establishing a consensus, whereas other studies often rely on the expertise of a single clinician [31,32].

Many studies have been published on building and using BNs as decision support tools; however, very few of them report the validation of such tools. In a review of prognostic models in obstetrics, only 8.7% of the tools were validated [30]. In using a comprehensive published framework, we have taken this a step further in establishing clinical confidence in the model (and areas that require further refinement), which will be required if the BN is to be used in clinical practice [33,34].

### Clinical Considerations

The results presented are promising for improving the identification and management of SSP. It is currently considered that clinicians in primary care make diagnoses with approximately 90% accuracy [35]. The use of a CDSS is intended to enhance, not replace, the expertise of clinicians [8]. Even if this BN could help to improve the accuracy rate by only 5 percentage points, the number of people who experience the long-term consequences of missed pathology would be halved. The last estimate of National Health Service expenditure on missed CES alone was £185 million (US $233 million) over 10 years for litigation costs [36]. Reducing the cases of missed SSP could significantly reduce these costs. If the BN were successfully deployed in a CDSS, it could help to improve the recognition of these rare conditions and reduce missed diagnoses.

Reducing variation in care is a key facet of the National Health Service long-term plan [37]. LBP is a complex condition to manage, particularly because of the many available pathways, including, but not limited, to orthopedics, rheumatology, musculoskeletal triage services, physiotherapy, self-management, pain management, and neurosurgery. This BN, as part of a CDSS, can systematically help clinicians to identify which patients with LBP are at risk of SSP and require escalation to specialist services, reducing unnecessary waits for inappropriate services. This would, in turn, leave conservative management options open for those who are at low risk [6].

The validity and reliability of red flag questions, designed to identify SSP, have been questioned in the recent literature [1,2,4,38]. Finucane et al [22] have opted to use a consensus method to provide a framework for the use of red flags in clinical practice. Our study used a similar methodology, recognizing the difficulty in bridging the gap between the lack of evidence for red flags and their regular use in clinical practice. By focusing on the SSP aspects of managing LBP in this study, we have prepared the model to consider safety in clinical use. In deploying this tool into a clinic, there is scope for machine learning from real-life data, thereby adding to the research knowledge about these difficult-to-manage cases.

Reducing unnecessary care using a CDSS has been proven in a wide range of health care settings. Compliance with guidelines represents reduction in unnecessary care such as overprescribing [39] and helps to manage waiting lists by ensuring that patients are referred to the correct service. The use of a CDSS has been shown to increase compliance with imaging guidelines in LBP [40], reduce prescription errors [41], improve the risk management of venous thromboembolism [42], and standardize cancer care in accordance with the evidence base [43]. These studies encompass a wide range of health care settings from community care to an intensive care unit. This study is the first step in developing a similar capability in SSP in LBP.

### Strengths and Limitations

The BN’s good performance, in particular on CES, inflammatory condition, and space-occupying lesion, is encouraging. In this regard, the elicitation process has been a success.

The erroneous results may be due to flaws in the elicitation process or the biases of the recruited clinicians. Certain risk factors identified in the literature were either not identified in this study or ranked too low to be included in the final model. A known issue with BNs is the need to constrain the number of variables and relationships to make the BN computationally viable [8]. This runs the risk of leaving out variables that a clinician may normally consider as part of the clinical reasoning process. However, the benefit of the Bayesian method is the combination of expert knowledge with research knowledge, as well as the ability to interrogate the model to explore these cases. This study underlines the need for transparency in decision-making and a combination of knowledge sources, both of which are possible with the Bayesian method.

Furthermore, the spinal experts involved in the validation were asked to recommend additional factors to be added to the model if they were judged to be missing. There was no consensus on any additional factors from the experts, perhaps reflecting the difficulty of assessing these pathologies, even for experienced clinicians.

This study provides a blueprint for the development of expert-elicited BNs and their validation. The elicitation procedure described here would be applicable for any musculoskeletal condition and, indeed, a wide range of medical conditions. In addition, this study goes a step further than previous development studies in demonstrating the utility and practicality of using a validation framework to assess the output of the elicitation and identify the areas of future development. This is a crucial next step toward building trustworthy BNs that have more chance of success in terms of adoption and implementation in health care.

### Next Steps

The next stage of development will involve building a web-based interface as a prototype CDSS knowledge-based AI system. It will need to be modified not only with regard to the clinical information collected here but also with regard to how risks are presented to the clinician, what action to take, and how urgently. An example design pipeline is presented in Figure 1. Further work on the usability and acceptability of the tool will be required to develop an interface that could put this information to best use in clinical practice. A mechanism to report outcomes to enable the AI learning component will be required. Further work to develop its capability to assess the optimum treatment for LBP that is not SSP should also follow.

This knowledge-based AI CDSS is a further step forward in providing clinicians in practice with access to expert reasoning. This method presents the combined reasoning of clinical experts, validated by the evidence base. The use of a BN as part of a CDSS enables expert recommendations to be made to patients from their first contact in the care pathway.

### Conclusions

This study has demonstrated that knowledge elicitation from multiple clinical experts was an effective way of developing a BN for complex decision-making within the constraint of a lack of structured data. The key positive features are that this was a systematic elicitation of complex information, it involved multiple expert participants, it led to consensus, and its results are largely consistent with independent expert judgment.

The resultant BN represents a model of clinical reasoning regarding SSP, with the potential capacity to incorporate machine learning and improve during use. The use of a validation framework has yielded important insights into areas where the BN has high levels of accuracy and areas that require further development. The structure and content of the BN have been independently reviewed with high levels of agreement between the study participants and the independent reviewers. The validation of the BN’s predictive ability highlighted issues with particular pathologies such as nerve root condition and fracture. The concurrent validation using research literature has provided well-defined avenues for improving the predictions that currently do not provide adequate accuracy.

Having the ability to identify SSP in LBP is a promising first step on the way to developing a BN for LBP as a whole. With this essential element in place, the elicitation process should be rerun to build models designed to guide the active management decisions for the mechanical and psychosocial aspects of LBP.

A prototype CDSS will be built using the BN to be used in a time frame appropriate for clinical consultations and be ready for testing in a clinical setting to guide further development.

## Appendix

Multimedia Appendix 1 Detailed description of the Bayesian network construction method.

## Abbreviations

AI: artificial intelligence
AUC: area under the curve
BN: Bayesian network
CDSS: clinical decision support system
CES: cauda equina syndrome
LBP: low back pain
NICE: National Institute for Health and Care Excellence
SSP: serious spinal pathology

## Data Availability

The data sets generated and analyzed during this study are available from the corresponding author on reasonable request.
