# Supplementary material for: Assessing Serious Spinal Pathology Using Bayesian Network Decision Support: Development and Validation Study
Source: JMIR Form Res. 2023 Oct 3;7:e44187. doi: 10.2196/44187 (PMC10582804; doi:10.2196/44187)
Supplement: Multimedia Appendix 1 [file formative_v7i1e44187_app1.docx]

# Appendix – Method for eliciting and constructing the BN.

Bayesian Networks (BNs) are graphical models that represent joint probability distributions of their variables. The graphical structure of a BN is a directed acyclic graph (DAG) consisting of nodes representing variables, and edges representing direct relationships between those variables (Figure A1). DAG encodes conditional independence assumptions that enable the joint probability distribution of the variables in a compact and factorized way. When an edge connects a node A to a node B (A 🡪 B), A and B are called the parent and child of each other respectively. Each node has a local conditional probability distribution between the node and its parents that is often represented by a conditional probability table (CPT). Figure A1 shows a simple BN example. In order to build a BN model, nodes (termed “variables” or “factors” in our elicitation) and edges (“relationships”) of its DAG need to be defined based on variables of importance in the problem domain and direct causal relations between those variables. Afterwards, an CPT is defined for each node in the DAG.


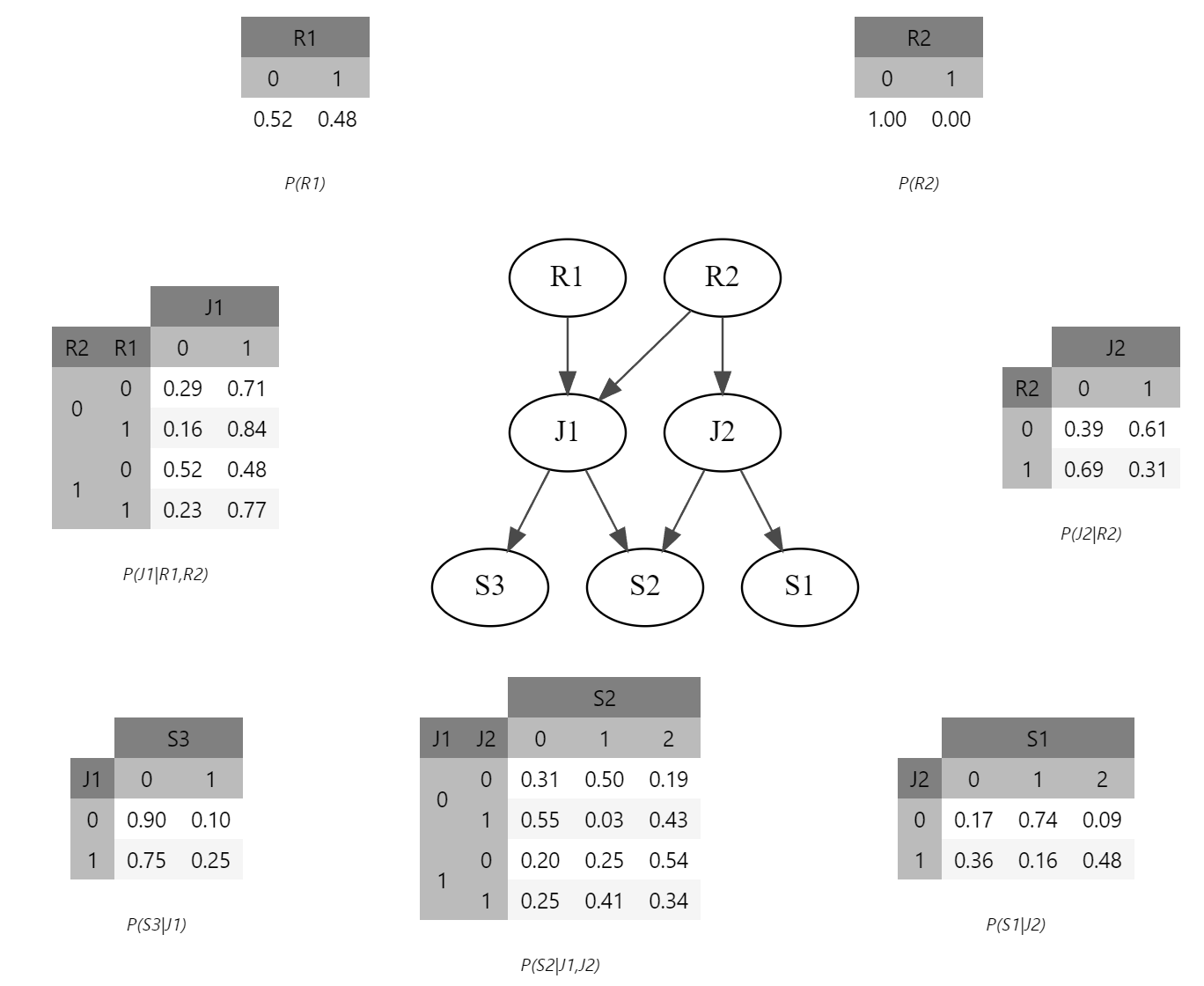


*Figure A1 Bayesian Network schematic, showing a directed acyclic graph (nodes and edges) representing , and the associated conditional probability tables for each node.*

Following the schematic in Figure A1, the elicitation process is composed of three stages

1. the *variables*,

2. their *structure* (i.e. direct) and

3. the associated conditional *probabilities*.

## Stage 1: Variables

Our BN model has three layers of variables, corresponding to the following three categories [8,16]:

1. *Risk factors:* These are factors that might raise (or lower) the chances of the patient having a certain condition. They may also have an effect on the efficacy of the treatment but, crucially, are factors (like age, or demographics) that cannot be changed by the treatment. These are represented by R1 and R2 in Figure A1.
2. *Judgement factors:* These might otherwise be known as clinical reasoning factors. They are the factors that are unlikely to be included directly in a PROM or test, but which help you reason about the prognosis or likelihood of recovery. These are represented by J1 and J2 in Figure A1.
3. *Signs and symptoms:* These are factors that arise as a consequence of the patient having certain conditions or judgement factors. In contrast to risk factors, signs and symptoms are factors that may change or respond to treatment. Some of these may also be considered as outcome measures for the patient. These are represented by S1, S2 and S3 in Figure A1.

We note that previous works [8,16] used the alternative terms *background factors*, *diseases* and *findings* respectively. However preliminary discussions [14] showed that the definitions above better reflected the clinical reasoning process in physiotherapy.

Stage 1 determines which variables are included in the model, and it was composed of an online and a workshop phase. In the online phase, participants were asked to populate an online interface (Figure 1) with variables that were clinically important to the patient’s assessment. Example variables were given, along with a clarifying question that could be answered in order to give the value of the variable (Table A1).

*Table A1: Example variable*

| Variable name | Variable states | Clarifying question |
| --- | --- | --- |
| Anxiety | High, medium, low | What is the patient’s anxiety level? |

In the online interface, participants had the ability to add new variables, and allocate variables to one of the described categories: Risk factors, Signs and symptoms, or Judgement factors. A Category Score (CS) were calculated for each variable based on the number of participants that place the variable in the highest voted category. For example, if 2, 3 and 7 participants place the variable in Risk Factors, Judgement Factors and Sign and Symptoms respectively then the CS is 7 out of 12 (or 58%) and the chosen category is Signs and Symptoms. Once the categories were allocated, participants ranked the variables in each category in order of importance.

In the workshop phase participants are presented with all the suggested variables (removing duplicates). Those achieving a Category Score of 80% or more were fixed in the associated category^[[1]](#footnote-1)^ and the rest placed in the ‘Available factors’ column. The ability to add more variables at this point was disabled. The variables in the ‘Available factors’ column were discussed with respect to categorisation and ranking, facilitated by a coordinator who used open-ended questions.

After the workshop discussion is completed, each participant allocates a category to each of the previously unallocated variables and updates the rankings in each category. Each variable was then given a CS and a Ranking Score (RS) to determine their final category and preference for model inclusion respectively. RS is a normalized ranking that is close to 0 if a variable is placed near the bottom and 1 if placed near the top, computed for every participant. RS of a variable is the median value of RS from all participants. The decision to include a variable in the model is then based on the RS, with an adjustment to favour cases where there is a consensus. Following the RAND Appropriateness Method recommendations [12], an 80% inter-percentile range (IPR) was used to give an indication of agreement. Smaller IPR values indicate greater consensus. The final decision to include into model was made based on an overall score (OS) that combines RS and IPR as shown below, where the $\alpha$ constant that determines the relative importance of consensus was set $\alpha=0.25$.

$$OS=RS-\alpha IPR$$

We estimated that around 50 variables could be taken forward without over burdening the participants in Stage 2 and reserved some clinical judgment to decide on the exact number. We selected 50 variables with the highest OS to the second stage.

## Stage 2: Structure

Stage 2 seeks to elicit the detailed structure of the BN by linking specific Risk Factors to Judgement Factors and Judgement Factors to Signs and Symptoms. Stage 2 was also composed of an online and a workshop phase. To obtain more statistical data we prefer a numerical value that characterises the ‘strength’ of every possible relationship rather than a simple yes/no existence elicitation. However information content is a difficult mathematical concept to non-experts and so we used qualitative descriptors, outlined in Table A2.

Table A2: Scoring definitions supplied to participants for Stage 2 elicitation.

| **Number** | **Definition** |
| --- | --- |
| 0 | No relationship |
| 1 | X *sometimes* has a *small* effect on Y |
| 2 | X *sometimes* has a *large* effect on Y **or** X *always* has a *small* effect on Y |
| 3 | X *always* has a *large* effect on Y |

Edges are only allowed from risk factors and judgement factors, and from judgement factors to signs and symptoms. This restriction is aligned with basic causal assumptions that risk factors influence the risk of clinical conditions (here termed “judgement factors”), and the clinical conditions (judgement factors) may manifest themselves as sign and symptoms. Moreover, it makes the elicitation of BN structure feasible by reducing the space of possible BN structures by highlighting the clinically plausible alternatives.

Every possible relationship within the restricted structure can be represented in the cells of a two-dimensional grid (see Figure 2). In the online phase, the participants judged the strength of every possible relationship using the strength descriptors in table A2. This was done in two grids; one covering links from risk factors to judgement factors (figure 2), and the other covering links from judgement factors to signs and symptoms. We used 80%-IPR across participants to measure the consensus of relationship strengths. Those relationships achieving an 80%-IPR of 1 or less were deemed to have sufficient consensus and were therefore fixed for the workshop phase.

In the workshop phase, the results of the online phase were discussed, with facilitation by the research team, as in Stage 1. The participants were asked to review their grids. Afterwards we used the OS equation described above to determine the highest strength relationships. A cut-off value is then used to create the BN structure by including all relationships above that value.

## Stage 3: Probability elicitation

The final stage elicited the CPTs associated to the variables in the causal model.

### CPT elicitation methods

In our discrete BN, the variables can either be binary, categorical or ordinal in nature. The CPT elicitation method used for a variable depends on the type of the variable.

For eliciting a binary variable, we need only ask the probability $p$ of one outcome (since the other is immediately $1-p$). In the online elicitation tool this corresponds to a single slider (see Figure A3). Eliciting small or large probabilities with sliders is problematic as a small differences can make higher impact. We scale the position of the slider with the following mapping (Figure A4) to provide a higher granularity for eliciting small and large probabilities.

$$T\left( x \right)=50-( 50\phi\left( \frac{1 - q^{\left( \frac{x}{50}- 1 \right)}}{1+ q^{\left( \frac{x}{50}- 1 \right)}} \right)+ 50), q = \frac{1- \psi}{1+\psi} , x \in[0, 100]$$

where $\psi=2\left( d-\frac{1}{2} \right)/\phi$, $\phi=1.02$ is a scaling factor, and $d=0.001$ is a minimum value to prevent 0 or 100 percentages.


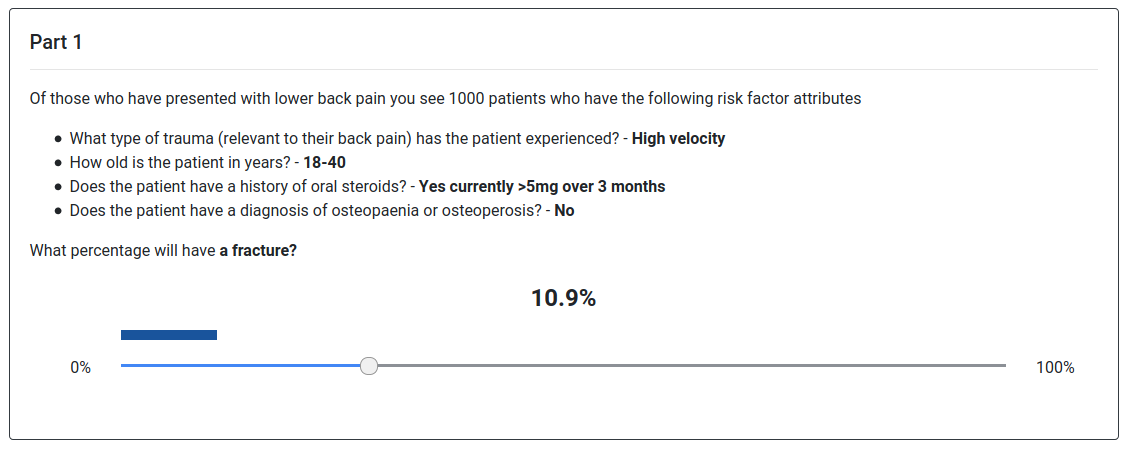


*Figure A3: Eliciting a binary probability for one combination of states of parent variables. This is an example of just one of the questions presented to the participants undertaking the probability elicitation*


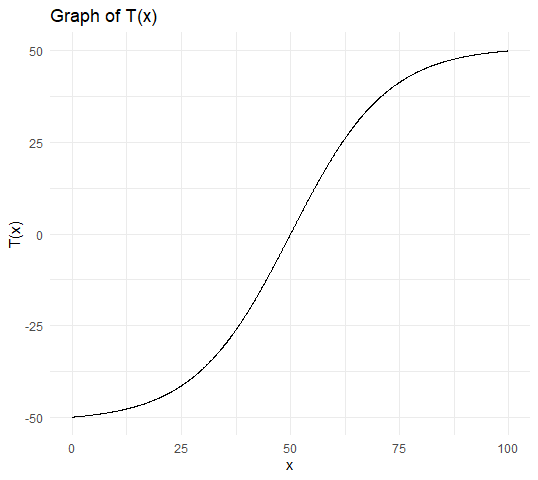


*Figure A4: Mapping function for ease of eliciting small and large probabilities, where x denotes the slider position and T(x) represents the indicated percentage.*

For a categorical variable, we must include sliders for all respective states (Figure A5) as adjusting one does not uniquely define any of the others (as it does in the binary case). To define a probability distribution, the probabilities must add up to 100%: If the values given do not then normalisation is used.


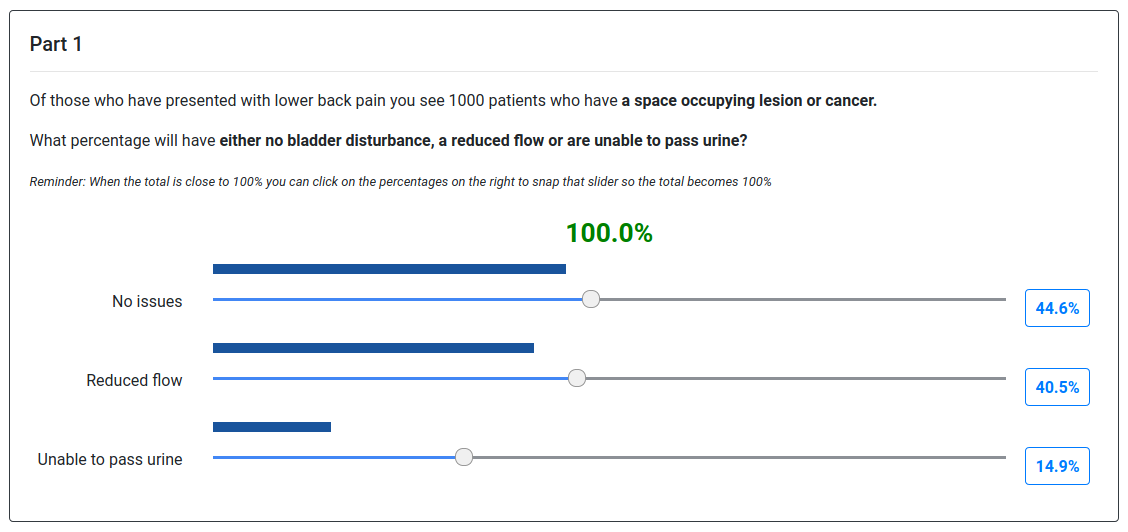


*Figure A5: Eliciting a probability for a categorical variable.*

Finally, we elicit the CPT for an ordinal variable. An ordinal variable represents a factor which has an inherent order or ranking in a natural progression e.g. low, medium, high. To model an ordinal variable, we make an approximation based on the Normal distribution that helps to avoid overfitting and reduces the number of elicitation parameters. The parameters $\mu$ and $\sigma$ that control the distribution are given the more intuitive names ‘Most likely value’ and ‘Degree of variation’ respectively (Figure A6). A visual representation of the distribution over the states is also provided.


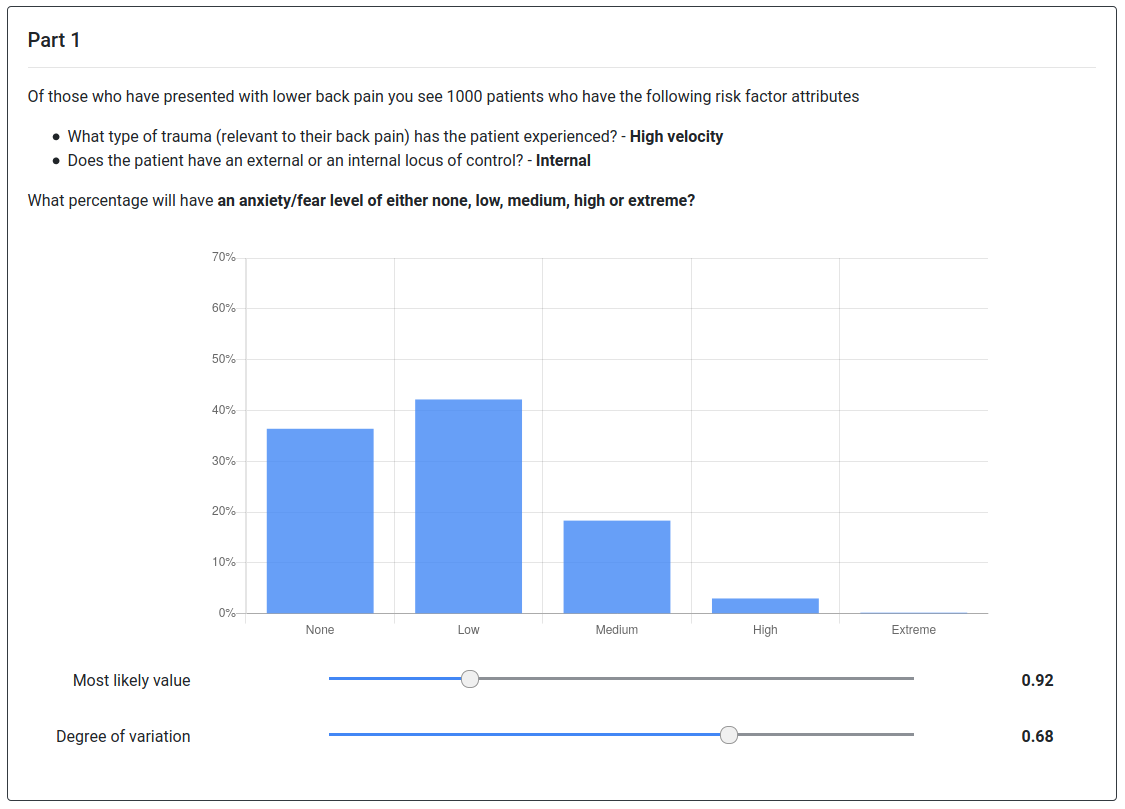


*Figure A6: Eliciting probability for an ordinal variable. This is just one combination of states of parent variables. In this example, a person’s level of anxiety or fear could be low, medium, high or extreme. The study participant selects the most likely value from these progressions, and then can adjust the degree of variation around that most likely value using the sliders.*

### CPT elicitation with parents

An unconditional variable with no parents has the equal number of probabilities as its states. Hence, its CPT can be elicited by using the elicitation methods shown in Figures A3, A4 or A5 only once. Eliciting CPTs of variables with parents is more challenging as their size grows exponentially with the number of parents, so using suitable approximations is the only reasonable alternative. We believe the natural choices are regression-type and Noisy-OR approximations, which reflect arguments from previous works [8,16] that the prevalence of diseases often depends on a linear combination of background/risk factors, whereas findings/signs & symptoms arise because one or other disease is present.

The study team took the decision to categorize the relation between variables into either ‘AND’ or ‘OR’ types. The former were variables where we believed the conditional probabilities of a positive result would be increased by linear combinations of the parents whereas the latter were situations where we believed the conditional probability was most likely to be raised by having one or other of the parents begin positive. Judgement factors were most likely to be AND variables and were modelled with a regression type approach, the OR variables were mostly signs and symptoms and were modelled by a Noisy-OR approximation (Figure A7).

*Figure A7: BN Structure. Prior, AND and OR variables are shown by circle, diamond and square nodes respectively. Binary, categorical and ordinal nodes are represented by red, blue and green nodes respectively.*

### AND variables

The CPT of AND variables were defined based on the type of the child variable. For categorical and binary variables, CPT was described by logistic regression.

$$P\left( Y=1 \mid X_{1}=x_{1},\ldots,X_{i}=x_{i}, ,\ldots,X_{n}=x_{n} \right)=\sigma\left( \beta\cdot\boldsymbol{x} \right)$$

where $\sigma$ is the sigmoid function and $\beta\cdot\boldsymbol{x}$ is expanded in the following manner

$$\beta_{0}+\sum_{i\mathcal{\in O,B}} \beta_{i}x_{i}+\sum_{i\mathcal{\in C}} \sum_{j=1}^{n_{i}} \beta_{i,j}x_{i}^{j}$$

in which $\mathcal{O,B}$ and $\mathcal{C}$ denote the sets of ordinal, binary and categorical parents of the variable $Y$ respectively and $n_{i}$ is the number of states of variable $X_{i}$. During elicitation, we show users different instantiations of $X$ and elicit probability values form them using the interfaces shown in Figure 3 and Figure 4. We estimate $\beta$ parameters based on these values provided by the user, and built the whole CPT based on the logistic regression model.

For ordinal variables, we follow a similar structure except the distribution is given by the Normal approximation. The mean and variance of an ordinal variable with parents is described by $\mu=\beta^{\mu}\cdot x$ and $\sigma^{2}=\beta^{\sigma}\cdot x$. In this case the parameters $\mu$ and $\sigma^{2}$ are elicited directly (see e.g. Figure [5](#fig: ranked elicitation)) rather than probabilities themselves as is the case for binary/categorical variables.

Since the probability elicitation process was voluntary, we could not guarantee the number of participants that would take part. For this reason we made a decision that a single completed process by one of the participants would generate enough information to create the CPTs for our BN. In the case of the risk variables, only one question each was needed due to the way the interface was constructed (see Figures A3, A5 and A6 for a question example for binary, categorical and ordinal variables respectively). For the AND variables it was therefore necessary to ask questions equal to the number of free parameters in this model. Various mechanisms for choosing this question set were discussed. For instance, having the same question set for each participant would facilitate a better analysis of potential consensus, however, ultimately, we decided upon what we believed the simplest solution, which was to randomly generate the question by choosing the parent states uniformly, subject to the linear-independence condition. The answers were then combined using regression methods.

### OR variables

OR variables are modelled using a noisy-OR approximation which is a generalization of logical OR gates [17]. The original Noisy-OR model assumes that effects of the parents on a binary child node are independent. Each parent *i* has a probability $p_{i}$ of causing the child node to be true. A leak factor $l$ represents the probability that the child node is true when all parents are false. For example, for a variable $Y$ with $n$ parents $X=\left( X_{1},\ldots,X_{n} \right)$, all of which takes values of either 0 or 1, a Noisy-OR model defines the CPT of $Y$ by

$$P\left( Y=1|X=x \right)=1-{(1-l)}^{\prod(1-x_{i})}\prod_{i=1}^{n} \left( 1-p_{i} \right)^{x_{i}}$$

where $p_{i}=P\left( Y=1|X_{1}=0,\ldots,X_{i}=1,\ldots,X_{n}=0 \right)$.

The generalisation of the Noisy-OR model to include multiple states has been investigated in [18-20]. To generalise the output variable $Y$ to multiple states (and likewise for each $\tilde{Y}_{\alpha}$) is straightforward, provided we assume there is a dominance in the state ordering. Specifically the states can be numbered $y=0,\ldots,s_{y}-1$ with an interpretation that an observation of $y$ includes all observations $y'<y$ (the state $y=0$ corresponds to a lack of observation). For example, the state *Bladder disturbance* has the states ($0=$ ‘No bladder disturbance’, $1=$ ‘Reduced flow’, $2=$ ‘Unable to pass urine’) since ‘Unable to pass urine’ would imply a ‘Reduced flow’. Noisy-OR model also extends to parent variables with multiple states.

For the elicitation we elicited the probabilities $P\left( Y \mid X_{i} \right)$ for all parents. Both binary/categorical and ordinal nodes were then elicited in the usual way, except that we didn’t need to elicit $P\left( Y \mid X_{i}=0 \right)$as this came from the noisy-OR assumption.

After completing the probability elicitation the answers are combined to form the final BN [15] (Figure 4).

1. They are also coloured differently for convenience. [↑](#footnote-ref-1)
